# Molecular simulations of conformation change and aggregation of HIV-1 Vpr13-33 on graphene oxide

**DOI:** 10.1038/srep24906

**Published:** 2016-04-21

**Authors:** Songwei Zeng, Guoquan Zhou, Jianzhong Guo, Feng Zhou, Junlang Chen

**Affiliations:** 1 School of Information and Industry, Zhejiang A & F University, Lin’an 311300, China; 2 Key Laboratory of Chemical Utilization of Forestry Biomass of Zhejiang Province, School of Sciences, Zhejiang A & F University, Lin’an 311300, China; 3 Zhe Jiang province environmental radiation monitoring center, Hangzhou 310012, China

## Abstract

Recent experiments have reported that the fragment of viral protein R (Vpr), Vpr13-33, can assemble and change its conformation after adsorbed on graphene oxide (GO) and then reduce its cytotoxicity. This discovery is of great importance, since the mutation of Vpr13-33 can decrease the viral replication, viral load and delay the disease progression. However, the interactions between Vpr13-33 and GO at atomic level are still unclear. In this study, we performed molecular dynamics simulation to investigate the dynamic process of the adsorption of Vpr13-33 onto GO and the conformation change after aggregating on GO surface. We found that Vpr13-33 was adsorbed on GO surface very quickly and lost its secondary structure. The conformation of peptides-GO complex was highly stable because of π-π stacking and electrostatic interactions. When two peptides aggregated on GO, they did not dimerize, since the interactions between the two peptides were much weaker than those between each peptide and GO.

Graphene oxide (GO) is a versatile derivative of graphene, functionalized with oxygen-contained groups^1^,^2^,^3^. Because of its water solubility, large specific surface area and functional groups, GO possesses strong physisorption ability and serves as an ideal substrate for adsorbing biomolecules without any surface modification^4^,^5^,^6^. GO presents growing potential in biomedical applications, such as enzyme immobilization^7^,^8^,^9^,^10^, drug delivery^11^,^12^,^13^ and biosensors^14^,^15^,^16^,^17^,^18^,^19^. For example, graphene-peptide complex could monitor the protein-peptide interactions^20^. Due to the π-π stacking and hydrophobic interactions, the pyrene-labeled peptide was strongly adsorbed on GO surface. The preferential adsorption of single-stranded DNA over double-stranded DNA on GO was also observed *via* π-π stacking and electrostatic interactions^18^,^19^,^21^,^22^.

Therefore, understanding the interactions between biomolecules and GO is fundamentally essential, especially for drug- and disease-related peptides or proteins. One such peptide or protein is virus protein R (Vpr), which is a small nuclear accessory protein of HIV-1^23^. The segment of Vpr, Vpr13-33, plays an important role in regulating nuclear importing of HIV through ion channel^24^. Recent scanning tunneling microscopy and circular dichroism studies have shown that Vpr13-33s aggregate on GO accompanied by conformation change from α-helix to β-sheet^25^. However, the atomic level information about the peptides-GO complex is largely unknown. Molecular dynamics (MD) simulations can thus be used to provide detailed information on the interactions between peptides or proteins and carbon nanoparticles. For example, Sun *et al*. have successfully explained the mechanism of the activity of α-chymotrypsin inhibited by GO using MD simulations^26^.

In this paper, we used all-atom MD simulations to investigate the conformation change and aggregation of Vpr13-33 on GO. Vpr13-33 was adsorbed on GO surface very quickly and then bent into U-shape. Both π-π stacking and electrostatic interactions contributed to the binding of Vpr13-33 on GO. When multi-peptides interact with GO, we observed that the peptides could assemble on GO surface with lower root-mean-square deviation (RMSD) because of steric effect.

## Computational Methods

The native structure of Vpr13-33 with sequence of EPYNEWTLELLEELKSEAVRH was download from Protein Data Bank (PDB code: 1FI0) and modeled by the AMBER03 force field^27^. The peptide was capped by acetyl and amine groups to avoid a possible salt bridge formed between the termini. GO were constructed based on a molecular formula of C_10_O_1_(OH)_1_(COOH)_0.5_ (i.e., 2 epoxy, 2 hydroxyl on both sides of graphene basal plane, and 1 carboxyl group on the edges of graphene, per 20 carbon atoms), which reflects a typical outcome of a standard oxidation process^28^,^29^. The hydroxyl and epoxy groups were randomly attached to both sides of the basal plane, and the carboxyl groups were also attached to the carbon atoms on the edge randomly. The unoxidized carbon atoms in GO were treated as uncharged Lennard-Jones (LJ) balls with a cross section of σ_cc_ = 3.4 Å and a depth of the potential well of ε_cc_ = 0.086 kcal/mol, and were restrained by a harmonic potential with a spring constant 2.4 kcal mol^−1^ Å^−2^ during the simulation^30^. The bonded parameters of carbon atoms in graphene were taken from Patra *et al*.^31^. The parameters of hydroxyl, carboxyl and epoxy groups were taken from the AMBER03 force field for serine, glutamic acid and dialkyl ether, respectively.

All MD simulations were performed by using the Gromacs package 4.5.5 with periodic boundary conditions in all directions^32^,^33^. The particle-mesh Ewald method was used to calculate the long-range electrostatic interactions, whereas the vdW interactions were treated with smooth cutoff at a distance of 12 Å^34^,^35^. Water was represented by the TIP3P model^36^. After energy minimization, the solvated systems were pre-equilibrated by MD simulations for 500 ps at a constant pressure of 1 bar and a temperature of 298 K with Berendsen coupling methods^37^. Then, the center of mass (COM) of Vpr13-33 was released, and the production simulation continued to be performed in an NVT ensemble at 298 K for 500 ns. Data were collected every 1 ps.

The binding energy of Vpr13-33 on GO was computed from the potential of mean force (PMF) using umbrella sampling. First, we conducted steered MD simulation to pull Vpr13-33 far away from GO, which was fixed during the simulation, and then 30 configurations were generated along the z-axis direction (reaction coordinate). The z coordinates of COM distance between Vpr13-33 and GO in each configuration differed by 0.1 nm. Each window was equilibrated for 5 ns and a production run of 5 ns was continued for sampling. Eventually, the PMF profile was obtained by the Weighted Histogram Analysis Method (WHAM), implemented in the GROMACS package as ‘g_wham’^38^.

## Results and Discussion

### Single Vpr13-33 on GO surface

**Figure 1:**
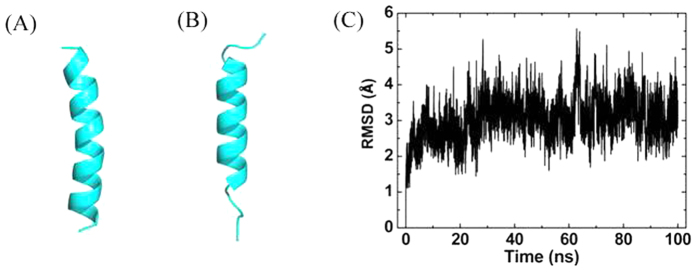
Vpr13-33 in the pure water. (**A,B**) The initial and final structures of Vpr13-33. (**C**) Time evolution of the RMSD of the backbone of Vpr13-33.

Prior to investigating the adsorption of Vpr13-33 on GO, we performed independent simulation to test the structural stability of the peptide in pure water, and the results were presented in Fig. 1. The secondary structure of the peptide is an α-helix, obtained from its crystal structure. Fig. 1B showed the snapshot at *t* = 100 ns. The two termini of the α-helix were destroyed, since they were more flexible. And the middle of the helix was kept intact. The RMSD of the backbone fluctuated at 3 Å, meaning that the secondary structure of Vpr13-33 remained well in the water.

**Figure 2:**
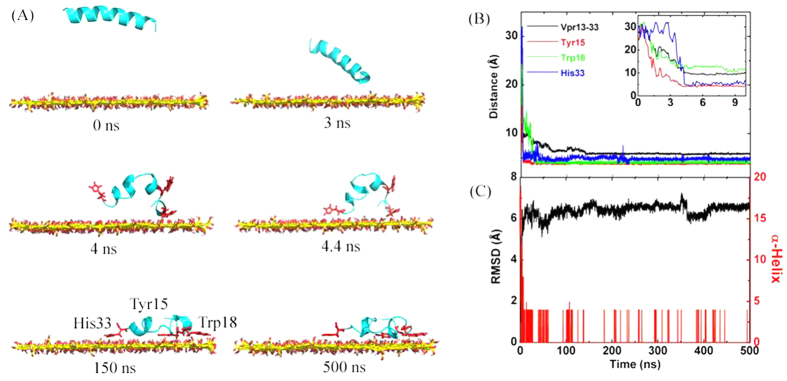
A representive trajectory of the adsorption of Vpr13-33 onto GO. (**A**) Side view of snapshots at critical time points. Vpr13-33 was depicted as a cartoon in cyan, but Tyr15, Trp18 and His33 specifically in red. (**B**) COM Distance between Vpr13-33, Tyr15, Trp18 and His33 and GO along the direction vertical to GO. Subfigure specified the distance in the first 10 ns. (**C**) The RMSD of Vpr13-33 from its native structure and the number of α-helical residues.

We then simulated the conformational dynamics of single Vpr13-33 on GO surface. GO was 37 × 54 Å^2^ in size. GO was put at the edge of the 50 × 65 × 65 Å^3^ of the box with its basal plane parallel to *xy*-plane. The COM distance between Vpr13-33 and GO was initially set as 27.5 Å, and the backbone of the peptide was parallel to the GO surface, as illustrated in Fig. 2A, *t* = 0 ns (water was not shown). Immediately, the N terminal residue moved downward to the GO surface (see Fig. 2A *t* = 3 ns). At *t* = 4 ns, another terminal residue was pulled down with COM distance between Vpr13-33 and GO fast decreasing from initial 30 Å to 9 Å (see Fig. 2B). Meanwhile, the peptide was deformed into U-bend and the whole α-helical structure was broken, accompanied by the RMSD of Vpr13-33 backbone straight climbing to 6 Å, 3 Å bigger than that in the pure water, and the number of residues in α-helix declining quickly to 4, as shown in Fig. 2C. This conformation change was induced by strong interactions between the termini of Vpr13-33 and GO. Then, the peptide continued to approach the GO surface to enhance their interactions, and unfolded partially on GO’s surface. Correspondingly, the COM distance between Vpr13-33 and GO gradually declined to the converged value at 6 Å after *t* = 150 ns. However, the RMSD had no distinct change but fluctuated slightly until the simulation was finished, and the number of α-helical residues just alternated between 0 and 4, implying that the conformation of Vpr13-33 on GO surface was highly stable.

**Figure 3:**
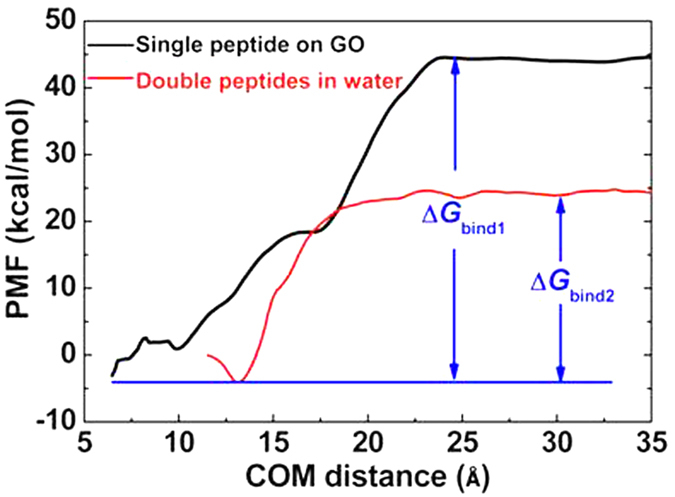
PMF profiles for the binding energy of Vpr13-33 on GO surface (black) and double peptides in the pure water (red).

The fast adsorption was driven by the strong attraction between Vpr13-33 and GO, which could be depicted by the binding energy (Δ*G*_bind1_). As shown in Fig. 3, the binding energy was increasingly higher when the peptide was far away from the GO surface. When the COM distance was more than 25 Å, their interactions were negligible. The value of Δ*G*_bind1_ was close to −50 kcal/mol. Therefore, once the COM distance was less than 25 Å, the peptide could continue to approach GO until tightly adsorbed on its surface.

To better understand the interactions between Vpr13-33 and GO, we found that there were three hydrophobic π-π structures formed between Tyr15, Trp18 and His33 of the peptide and GO, and the aromatic or heterocyclic rings were in the flat mode (see Fig. 2A, *t* = 150 ns). We then calculated the distances between the COM of the above three residues and GO (see Fig. 2B). Till *t* = 4.4 ns, the distances between Tyr15, His33 (which are located on or near the termini of Vpr13-33) and GO reached about 4 Å successively, and stayed at this value until the end of simulation, indicating that the two π-π structures were the main forces to keep the peptide U-shape.

**Figure 4:**
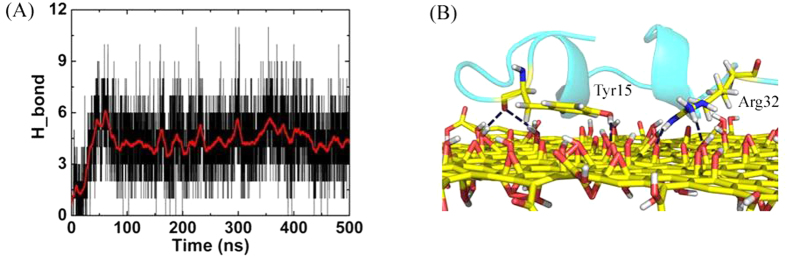
Electrostatic interaction between Vpr13-33 and GO. (**A**) The number of hydrogen bond between Vpr13-33 and GO. (**B**) Snapshot at *t* = 497.9 ns, the blue dash lines represent the hydrogen bonds.

Since there are a large number of oxygen-contained groups on GO surface, electrostatic interaction is another important force contributing to the binding affinity of Vpr13-33 on GO. We then analyzed the number of hydrogen bond formed between the peptide and GO, as illustrated in Fig. 4A. The hydrogen bond was sensitive to the position of each atom, therefore, the number fluctuated dramatically because of thermal motion. As average, there were about 5 hydrogen bonds between the peptide and GO after equilibrium. For example, there were 5 hydrogen bonds in Fig. 4B, with 3 between Tyr15 and GO, and 2 between Arg32 and GO, respectively.

The previous studies have revealed that the unfolding of α-helical peptides after adsorbed on graphene is induced by the strong vdW and hydrophobic interactions^39^, while electrostatic interaction and steric effect prevent the peptide from further unfolding^40^. On the contrary, electrostatic interaction enhances the stability of the binding of proteins on GO. Therefore, Vpr13-33 had no essential conformation change and the RMSD as well as the α-helical residues only fluctuated slightly after adsorbed on GO, as shown in Fig. 2C.

### Aggregation of double Vpr13-33s on GO

**Figure 5:**
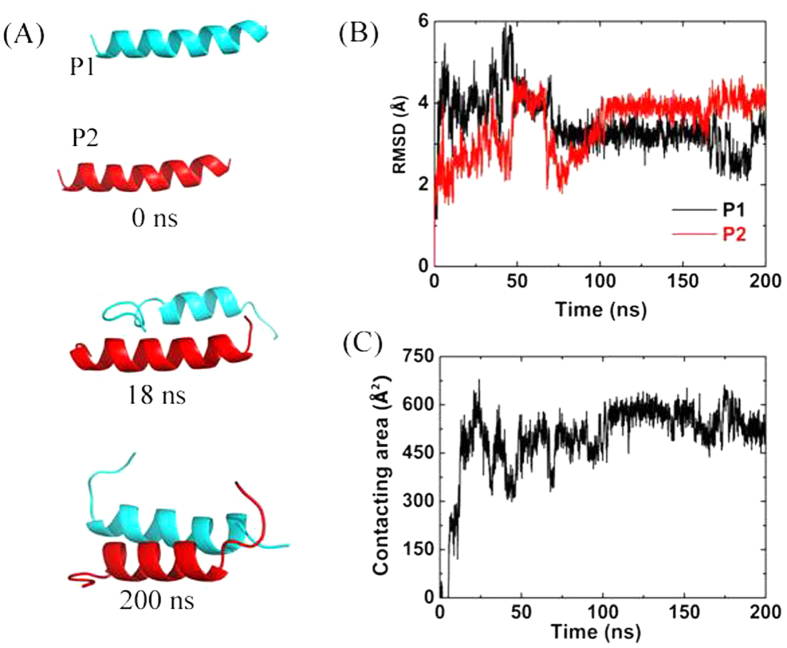
Aggregation of double peptides in pure water. (**A**) Snapshots taken at 0 ns, 18 ns, 200 ns. (**B**) The RMSDs of the two peptides. (**C**) Contacting area between the two peptides.

Similarly, we first simulated the aggregation of two peptides in the water. Here, we employed a new parameter, contacting surface area (CSA), to characterize the dimerization, as shown in Fig. 5C. The CSA was defined by the following formula:


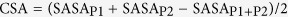


where SASA represents the solvent accessible surface area. Initially, the CSA was zero, since the two peptides were far away and the COM distance between them was set as 25 Å (see Fig. 5A, *t* = 0 ns). Then, the CSA rose fast to about 600 Å^2^ at *t* = 18 ns, indicating that the two peptides had dimerized. Just like single peptide in the water, the two termini of the peptides unfolded and the middle helixes were maintained well. Correspondingly, the RMSDs of the two peptides fluctuated at 4 Å.

**Figure 6:**
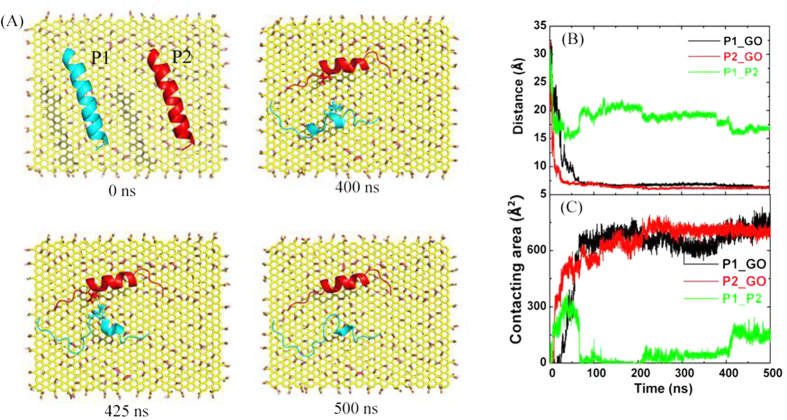
The aggregation of double peptides on GO. (**A**) Top view of snapshots taken at 0 ns, 400 ns, 425 ns and 500 ns. The two peptides were labeled as P1 (cyan) and P2 (red), especially, Leu22 in P1 and Leu20 in P2 were shown as sticks. (**B**) COM Distance and (**C**) contacting surface area among the two peptides and GO.

To investigate the aggregation of Vpr13-33 on GO, we put two peptides in the simulation box and enlarged the size of GO with 55 × 68 Å^2^. The COM distances between the two peptides and GO and between the two peptides themselves were the same 25 Å (see Fig. 6A, *t* = 0 ns). The two peptides went to GO surface separately and were finally adsorbed on GO, since the binding energy of Vpr13-33 on GO (Δ*G*_bind1 _~ −50 kcal/mol) was much stronger than that of double Vpr13-33 s in the water (Δ*G*_bind2_ ~ −30 kcal/mol, see Fig. 3). Figure 6 showed one typical trajectory. Since GO possessed large surface area to adsorb biomolecules, the two peptides had enough space to extend after adsorbed on GO surface. Therefore, the COM distances between peptides and GO were close to that of single peptide-GO system, about 6 Å.

The CSAs between each peptide and GO were alike with each other (Fig. 6C), about 700 Å^2^ after equilibrium. However, the CSA between the two peptides was only 150 Å^2^, meaning that the two chains were not dimeric and the interactions between peptides were much weaker than that between each peptide and GO. The two peptides could dimerize in the pure water (see Fig. 5A) or on pristine graphene (PG) (see Figure S1 in the Supplementary Information). Because of the smooth PG surface, peptides could slide on it and interpeptide hydrophobic interactions compel the peptides to form a dimer. In GO system, electrostatic interaction and steric effect that originate from oxygen-contained groups on GO surface enhanced the stability of the adsorption and hindered the peptides from sliding freely. Therefore, the two peptides exhibited no obvious dimerization. However, interpeptide hydrophobic interactions could still make the two peptides approach further. As shown in Fig. 6C, the CSA between the two peptides had a slight climb near *t* = 400 ns. This climb happened between Leu22 in P1 and Leu20 in P2 (see Fig. 6A, *t* = 425 ns), which are hydrophobic residues.

**Figure 7:**
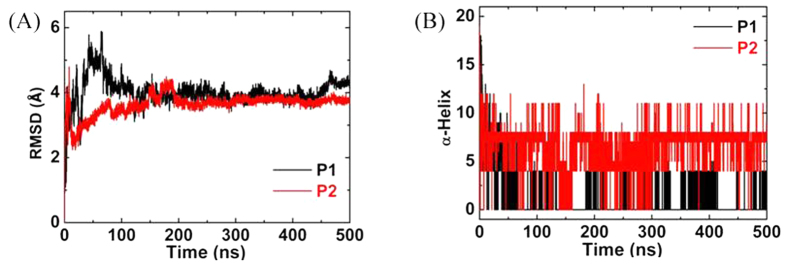
Conformational changes of the two peptides. (**A**) The RMSDs of backbone of P1 and P2. (**B**) The number of residues in the α-helix structure.

Both peptides lost their partial secondary structures after adsorbed on GO surface. The RMSDs of the two peptides were close to 4 Å (see Fig. 7A), close to those in pure water, but 2 Å less than that of single peptide on GO surface, because the main chains of the two peptides did not bend. Comparing the adsorption of peptides, protein fragments and globular proteins on GO, we could speculate that the effect of GO on conformation change of peptides or proteins would be more and more weak with the increasing of chain length because of steric effect^26^,^40^.

## Conclusions

In summary, molecular dynamic simulations have been performed systematically to explore the adsorption of Vpr13-33 on GO. The simulation results confirm that GO can induce conformation change and aggregation of Vpr13-33. The conformation of Vpr13-33 on GO surface is highly stable via π-π stacking and electrostatic interactions, while electrostatic interactions and steric effect prevent Vpr13-33 further unfolding. Compared with the adsorption of peptides on pristine graphene, where two peptides are dimeric, the peptides are separately located on GO surface, since the interactions between each peptide and GO are much stronger than interpeptide hydrophobic interactions.

## Additional Information

**How to cite this article**: Zeng, S. *et al*. Molecular simulations of conformation change and aggregation of HIV-1 Vpr13-33 on graphene oxide. *Sci. Rep*. **6**, 24906; doi: 10.1038/srep24906 (2016).

## Supplementary Material

Supplementary Information
