# Supplementary material for: Molecular simulations of conformation change and aggregation of HIV-1 Vpr13-33 on graphene oxide
Source: Sci Rep. 2016 Apr 21;6:24906. doi: 10.1038/srep24906 (PMC4838942; doi:10.1038/srep24906)
Supplement: Supplementary Information [file srep24906-s1.doc]

Supplementary Information to

"Molecular Simulations of Conformation Change and Aggregation of HIV-1 Vpr13-33 on Graphene Oxide"

by

Songwei Zeng, Guoquan Zhou, Jianzhong Guo, Feng Zhou & Junlang Chen


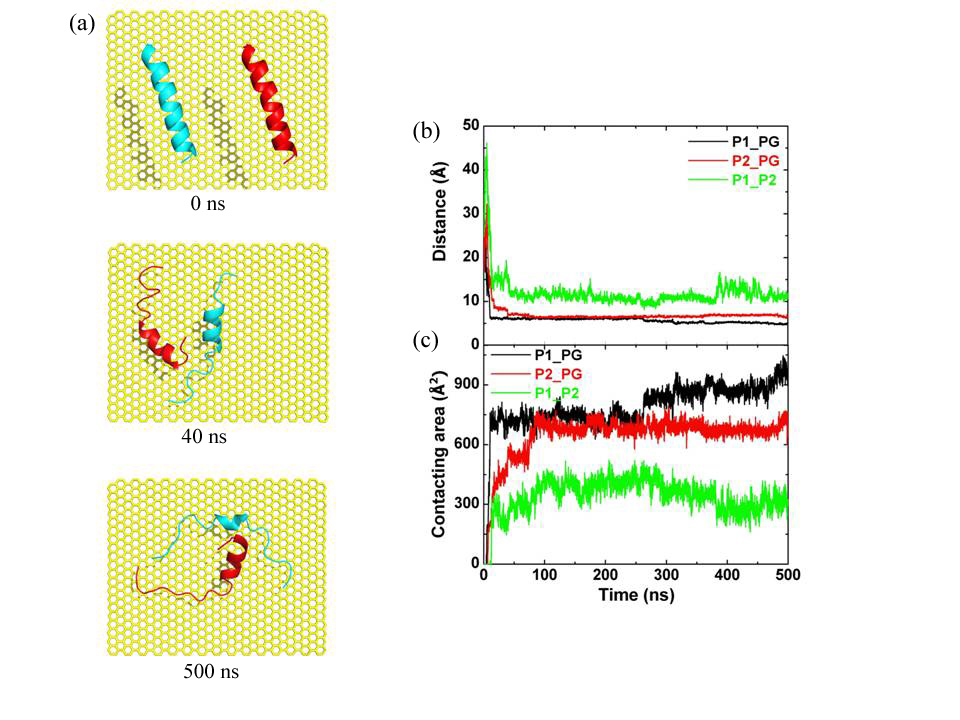


Figure S1. (Control run) Aggregation of double peptides on pristine graphene (PG). (a) Top view of snapshots taken at 0 ns, 40 ns, and 500 ns. (b) COM Distance and (c) contacting surface area among the two peptides and PG.

Figure S1 shows the trajectory of double peptides adsorbed on PG surface. Since the PG surface is much smooth, the binding sites of peptides on PG are not fixed and interpeptide hydrophobic interactions compel the peptides to form a dimer. The contacting surface area between the two peptides is about 300 Å^2^, which is twice as that on GO surface.
